# Uncovering Bupi Yishen Formula Pharmacological Mechanisms Against Chronic Kidney Disease by Network Pharmacology and Experimental Validation

**DOI:** 10.3389/fphar.2021.761572

**Published:** 2021-11-15

**Authors:** Difei Zhang, Bingran Liu, Xina Jie, Jiankun Deng, Zhaoyu Lu, Fuhua Lu, Xusheng Liu

**Affiliations:** ^1^ The Second Clinical College of Guangzhou University of Chinese Medicine, Guangzhou, China; ^2^ Department of Nephrology, Guangdong Provincial Hospital of Chinese Medicine, Guangzhou, China

**Keywords:** traditional Chinese medicine, bupi yishen formula, chronic kidney disease, network pharmacology, experimental study

## Abstract

Chronic kidney disease (CKD) is a leading public health problem with high morbidity and mortality, but the therapies remain limited. Bupi Yishen Formula (BYF) - a patent traditional Chinese medicine (TCM) formula - has been proved to be effective for CKD treatment in a high-quality clinical trial. However, BYF’s underlying mechanism is unclear. Thus, we aimed to reveal BYF pharmacological mechanism against CKD by network pharmacology and experimental studies. Network pharmacology-based analysis of the drug-compound-target interaction was used to predict the potential pharmacological mechanism and biological basis of BYF. We performed a comprehensive study by detecting the expression levels of fibrotic and inflammatory markers and main molecules of candidate signal pathway in adenine-induced CKD rats and TGF-β1-induced HK-2 cells with the treatment of BYF by western blotting and RT-qPCR analyses. Using small interfering RNA, we assessed the effect of BYF on the TLR4-mediated NF-κB mechanism for CKD renal fibrosis and inflammation. Network pharmacology analysis results identified 369 common targets from BYF and CKD. Based on these common targets, the BYF intervention pathway was analyzed by Gene Ontology (GO) and Kyoto Encyclopedia of Genes and Genomes (KEGG) enrichment analysis. We found that Toll-like receptor (TLR) and NF-κB signaling pathways were enriched. Then, we demonstrated that BYF significantly improved the adenine-induced CKD rat model condition by kidney dysfunction improvement and reversing renal fibrosis and inflammation. Subsequently, we investigated BYF’s effect on the TLR4/NF-κB signaling pathway. We found that TLR4 and phospho-NF-κB (p-p65 and p-IKβα) expression was significantly upregulated in adenine-induced CKD rats, then partially downregulated by BYF. Furthermore, BYF inhibited fibrotic and inflammatory responses, as well as TLR4, p-p65, and p-IKβα in TGF-β1-induced HK-2 cells. Additionally, the BYF inhibitory effect on fibrosis and inflammation, and NF-κB pathway activation were significantly reduced in TGF-β1-induced HK-2 cells transfected with TLR4 siRNA. Altogether, these findings demonstrated that the suppression of TLR4-mediated NF-κB signaling was an important anti-fibrotic and anti-inflammatory mechanism for BYF against CKD. It also provided a molecular basis for new CKD treatment drug candidates.

## Introduction

Chronic kidney disease (CKD) is a worldwide public health problem with an estimated global prevalence of 8–16% (Jha, et al., 2013). CKD affects approximately 8–10% of the Western countries population (Lameire, et al., 2005), and >15% of U.S. adults may have CKD. In China, the total CKD prevalence is around 10.8% and affects more than 120 million individuals (Zhang, et al., 2012). Also, CKD is associated with adverse cardiovascular events and high mortality risk (Bello, et al., 2011). However, there are limited treatment options available for CKD patients. The main approach to delay CKD progression is renin-angiotensin system (RAS) blockade, as well as blood pressure and glycemic control (Palmer, et al., 2015; Breyer and Susztak, 2016). These therapeutic strategies are insufficient to impair CKD progression to end-stage renal disease (ESRD). Therefore, it is urgent to develop effective medications to prevent CKD progression.

Traditional Chinese medicine (TCM) is commonly used in conjunction with Western medications for CKD treatment in China and other Asian countries (Li and Wang, 2005; Wojcikowski, et al., 2006; Zhong, et al., 2013). However, the use of TCM in CKD treatment remains controversial, especially because of renal toxicities present in some TCM (Feng, et al., 2018; Yang B et al., 2018; Omer Mohamed, et al., 2020). There is also emerging solid evidence of the beneficial effects of TCM prescribed for CKD patients in mainland China (Zhang, et al., 2014; Li, et al., 2020; Wu, et al., 2021) and Taiwan (Lin, et al., 2015), supporting that TCM can be promising for the development of new therapeutic drugs for CKD treatment.

The Bupi Yishen Formula (BYF), a patent TCM, is composed of eight herbs, which are modified from one traditional Si-Jun-Zi Decoction (SJZD). SJZD was recorded in Tai Ping Hui Min He Ji Ju Fang (A.D.1078-1,110) and is used to replenish “Qi” and reinforce “Spleen”. Based on TCM theory, BYF can “reinforce the Spleen and invigorate the Kidney” and “dispel dampness and resolve turbidity”, suggesting that it could be used for CKD treatment. Over the past decade, BYF has been clinically applied as a basic treatment for CKD patients. Our recently published multicenter randomized controlled trial demonstrated that BYF significantly improved kidney function in non-diabetic CKD4 patients, as evidenced by a slower decline slope of the estimated glomerular filtration rate (eGFR) and a lower composite outcome risk (Mao, et al., 2020). However, BYF’s underlying reno-protective mechanism remains unknown and requires investigation.

In this study, we first identified the BYF and CKD common targets. Then, we analyzed the intervention pathways based on these common targets using network pharmacology. Second, we examined BYF’s therapeutic effect on CKD *in vivo.* We used an adenine-induced CKD rat model and found that BYF reduced renal fibrosis and inflammation, and simultaneously inhibited the TLR4/NF-κb pathway. Finally, the BYF regulatory mechanism on renal fibrosis and inflammation was validated *in vitro* with TGF-β1-induced HK2 cells and TLR4 siRNA. Altogether, our study demonstrated that BYF reduced renal fibrosis and inflammation by TLR4-mediated NF-κb signaling pathway suppression, which may be a key mechanism of its therapeutic effect on CKD.

## Materials and Methods

### Active Compounds and Corresponding Drug Targets Collection

The BYF chemical compounds were screened using TCMSP (http://tcmspw.com/tcmsp.php) (Ru, et al., 2014), TCMID (http://www.megabionet.org/tcmia/), and BATMAN-TCM (http://bionet.ncpsb.org/batman-tcm/index.php.Home/Index/index). Compounds that showed DL ≥ 0.18 and OB ≥ 30% were retrieved as active by ADME analysis (Wang, et al., 2015). We collected active compounds targets with the TCMSP and SYMMAP (http://www.symmap.org) databases (Wu, et al., 2019), then retrieved the target’s gene name and ID using the Uniprot (https://www.uniprot.org/) database.

### Disease and Drug-Disease Common Targets Collection

The genes of targets related to “Chronic kidney disease” were screened *via* GeneCards (https://www.genecards.org/) (Stelzer, et al., 2011), OMIM (https://www.omim.org/) (Amberger, et al., 2015), TTD (http://db.idrblab.net/ttd/), MALACARDS (https://www.malacards.org/) (Rappaport, et al., 2017), and DisGeNET database (http://disgenet.org/) (Piñero, et al., 2017). After removing duplicated genes, the common targets associated with BYF and CKD were collected as the candidates.

### Drug-Compound-Target Interaction Network Construction

To analyze the relationship between drugs, active compounds, and candidate targets, a drug-compound-target interaction network was constructed using Cytoscape 3.6.1 software. In the network, different targets, active compounds, and drugs were represented as different colors and shapes nodes.

### Protein-Protein Interaction Network Construction

The String database contains a large number of PPI relationships (von Mering, et al., 2005). The candidate targets were unloaded onto the String database (https://string-db.org/) to obtain related information about protein interactions. Then, a PPI network was Constructed using Cytoscape 3.6.1 software. Besides, the median of three topological indexes was calculated (BC, CC, and DC), and the core PPI network targets were screened.

### Enrichment Analysis

Gene Ontology (GO) functional and Kyoto Encyclopedia of Genes and Genomes (KEGG) pathway enrichment analysis were performed based on the candidate targets using DAVID 6.8. The downloaded results were sorted using *p* and count values. The workflow used for this study was shown in Supplementary Figure S1.

### BYF Water Extract Preparation

BYF contains eight Chinese herbs. The related BYF herbal information and chemical composition analysis were performed as previously described (Zhang, et al., 2018; Mao, et al., 2020). Raw herbs were purchased from Jiangyin Tianjiang Pharmaceutical Co., Ltd. (Jiangsu, China). The eight BYF components, including Astragali radix (30 g), Codonopsitis radix (15 g), Atractylodis macrocephalae rhizome (12 g), Poria (15 g), Diosscoreae rhizome (15 g), Coicis semen (20 g), Cuscutae semen (12 g), and Salviae miltiorrhizae radix et rhizome (15 g), were boiled twice (1 h each) in ddH2O (w/v). The extract was condensed and stored at −20 °C. Before treatment, the BYF extract was dissolved in distilled water.

### Animals and Experimental Treatment

This animal experiment was performed according to protocols approved by the Institutional Ethics Review Boards of the Second Clinical College of Guangzhou University of Chinese Medicine, Guangzhou, China (approval No. 2020021). Twenty-four male Spraque-Dawley (SD) rats (∼200 g of weight) were purchased from the Guangdong Experimental Animal Center (Guangzhou, China). They were housed in the SPF animal breeding room with a 12-h light/12-h dark cycle and the temperature was maintained at 22–25°C. Rats were randomly divided into four groups (n = 6 for each group): 1) control (CTL); 2) untreated CKD (CKD); 3) CKD treated with BYF-Low dose (BYF-L); and 4) CKD treated with BYF-High dose (BYF-H). The CKD in rats was induced by intra-gastric gavage with adenine (Sigma-Aldrich, St Louis, MO, USA) at 200 mg/kg for 4 weeks (Chen, et al., 2017; Thakur, et al., 2018). Rats in the CKD treatment groups received BYF extract doses of 15 g/kg/d (BYF-L) and 30 g/kg/d (BYF-H) for 4 weeks with simultaneous adenine administering. Rats in CTL received normal adenine-free saline solution for 4 weeks.

### Biochemical Analysis of Serum and Urine Samples

Serum creatinine and urea, 24 h urinary protein, urinary albumin to creatinine ratio, aspartate transaminase (AST), and alanine transaminase (ALT) were measured using a Roche automatic biochemistry analyzer (Hitachi, 7180, Tokyo, Japan) following the manufacturer’s instructions. Analyses were performed in the Laboratory Department of the University City Hospital of Guangdong Hospital of Traditional Chinese Medicine.

### Histological Examination

Rat kidney samples were fixed with 4% buffered paraformaldehyde (pH 7.4) at 4°C overnight, dehydrated in graded alcohols, and embedded in paraffin. The paraffin-embedded kidneys were cut into 2 μm sections and stained with hematoxylin and eosin (HE), periodic acid-Schiff (PAS), and Masson’s trichrome for pathological changes evaluation. The tubular atrophy score was performed in PAS staining. The interstitial fibrosis was assessed by collagen deposition area in Trichrome staining using the ImageJ software (NIH, Bethesda, MD, United States).

### Immunohistochemical Analysis

For immunohistochemistry staining, 2 μm paraffin-embedded sections were deparaffinized and rehydrated. The antigens were repaired with 1% (w/v) Tris-EDTA solution by high temperature and pressure for 10 min. Sections were blocked with BSA, then incubated with primary antibodies. The following antibodies were used: TGF-β1 (3711; Cell Signaling Technology), α-SMA (19,245; Cell Signaling Technology), fibronectin (NBP1-91258, Novus), and TLR4 (sc-293072, Santa Cruz). The secondary antibody was horseradish-peroxidase (HRP) goat anti-rabbit IgG (J31126; Transgen Biotech).

### Cell Culture and Treatment

The HK-2 cells were cultured in DMEM/F12 supplemented with 10% FBS, 25 mM glucose, and 1% penicillin and streptomycin. They were incubated in a 37°C humidified incubator supplied with 5% CO_2_. The HK-2 cells were passaged using 0.25% Trypsin at a 1:6–8 ratio every 3–4 days. At 80% confluence, the cells were starved in 0.5% FBS overnight. Then, cells were divided into negative control (CTL), TGF-β1, and BYF groups. The TGF-β1 group was treated with 10 ng/ml TGF-β1 for 48 h. The BYF group was treated with 10 ng/ml TGF-β1 for 24 h, then treated with 32 mg/ml BYF for 24 h.

### Cell Viability

The CCK-8 assay was applied to assess the BYF effect on cell viability, following the manufacturer’s instructions. First, serum-starved HK-2 cells were treated with different BYF concentrations (0, 32, 64, 128, 188, 256, 375, 512, 750, 1,500 mg/ml) with or without TGF-β1 for 48 h. Second, 10 μl of CCK-8 reagent reacted with HK-2 cells at 37°C for 2 h. Finally, the supernatant was removed, and the optical density was measured at 490 nm using a microplate reader (TECAN, Mannedorf, Switzerland).

### TRL4 Downregulation by Small Interfering RNA

Transfection of siRNA was used to downregulate TRL4 in the HK-2 cells. The HK-2 cells were transfected with 10 μM siRNA targeting TRL4 (si-TRL4) or control siRNA (si-CTL) using Lipofectamine 2000 reagent (Invitrogen, Carlsbad, CA, United States), according to the manufacturer’s protocols. After siRNA transfection, cells were incubated with or without BYF for 48 h, with or without TGF-β1.

### RNA Extraction and qPCR Analysis

Total RNA was extracted from the kidney cortex and HK2 cells using TRIzol Reagent (Invitrogen, Carlsbad, CA). About 500 ng of extracted RNA was reversely transcribed to cDNA with the QuantiTect Reverse Transcription Kit. Then, cDNA samples were used to conducted real-time PCR analysis with an SYBR Green I Kit. Gene expression quantification was normalized to Glyceraldehyde-3-Phosphate dehydrogenase (GAPDH). The expression level fold change relative to the control group was calculated using the 2^-△△Ct^ method. The primers used for this study are described in Supplementary Table S1.

### Western Blotting Analysis

The kidney cortex and cultured cells were lysed in radioimmunoprecipitation assay (RIPA) buffer containing a protease inhibitor cocktail (Roche) and phosphatase inhibitor. Protein concentration was measured by a BCA detection kit (Thermo Fisher Scientific). The same protein amount (50 μg) was electrophoresed using 10% SDS-PAGE gels, then transferred to polyvinylidene difluoride membranes (Millipore, Billerica, MA, United States). The membranes were blocked with 5% nonfat milk for 1 h, then incubated with primary antibodies overnight at 4°C. Membranes were incubated with horseradish peroxidase (HRP)-conjugated anti-mouse IgG (Boster, BA1050) or HRP-conjugated anti-rabbit IgG (Boster, BA1054) for 1 h at room temperature. Their HRP activity was visualized using an enhanced chemiluminescence reagent (Bio-RAD, Bio-Rad universal Hood II, California, United States) and Image Lab System (Bio-RAD 5.2.1) was used for densitometric analysis. The primary antibodies used for this study included TGF-β1 (3711; Cell Signaling Technology); α-SMA (19,245; Cell Signaling Technology); fibronectin (NBP1-91258, Novus); Collagen I (NB600-408, Novus); Collagen III (NBP1-05119, Novus); Smad3 (9523S; Cell Signaling Technology); p-Smad3 (9520S; Cell Signaling Technology); TLR4 (sc-293072, Santa Cruz); NF-κb (p65, ab16502, Abcam); p-NF-κb (p-p65, 3033S, Cell Signaling Technology); IKβα (4814S, Cell Signaling Technology); p-IKβα (2859s, Cell Signaling Technology); and MyD88 (ab219413, Abcam).

### Statistical Analyses

Each analysis is representative of at least three independent repeats of experiment. GraphPad Prism five software (GraphPad Software Inc, La Jolla, CA) was used for statistical analysis. The data are represented as mean ± standard deviations (SD). Differences between two groups were analyzed using a 2-tailed Student’s t-test and two-way analysis of variance (ANOVA) was used for comparison between three or more groups. A *p*-value < 0.05 was considered statistically significant.

## Results

### BYF Active Compounds and Candidate Targets in CKD

**FIGURE 1:**
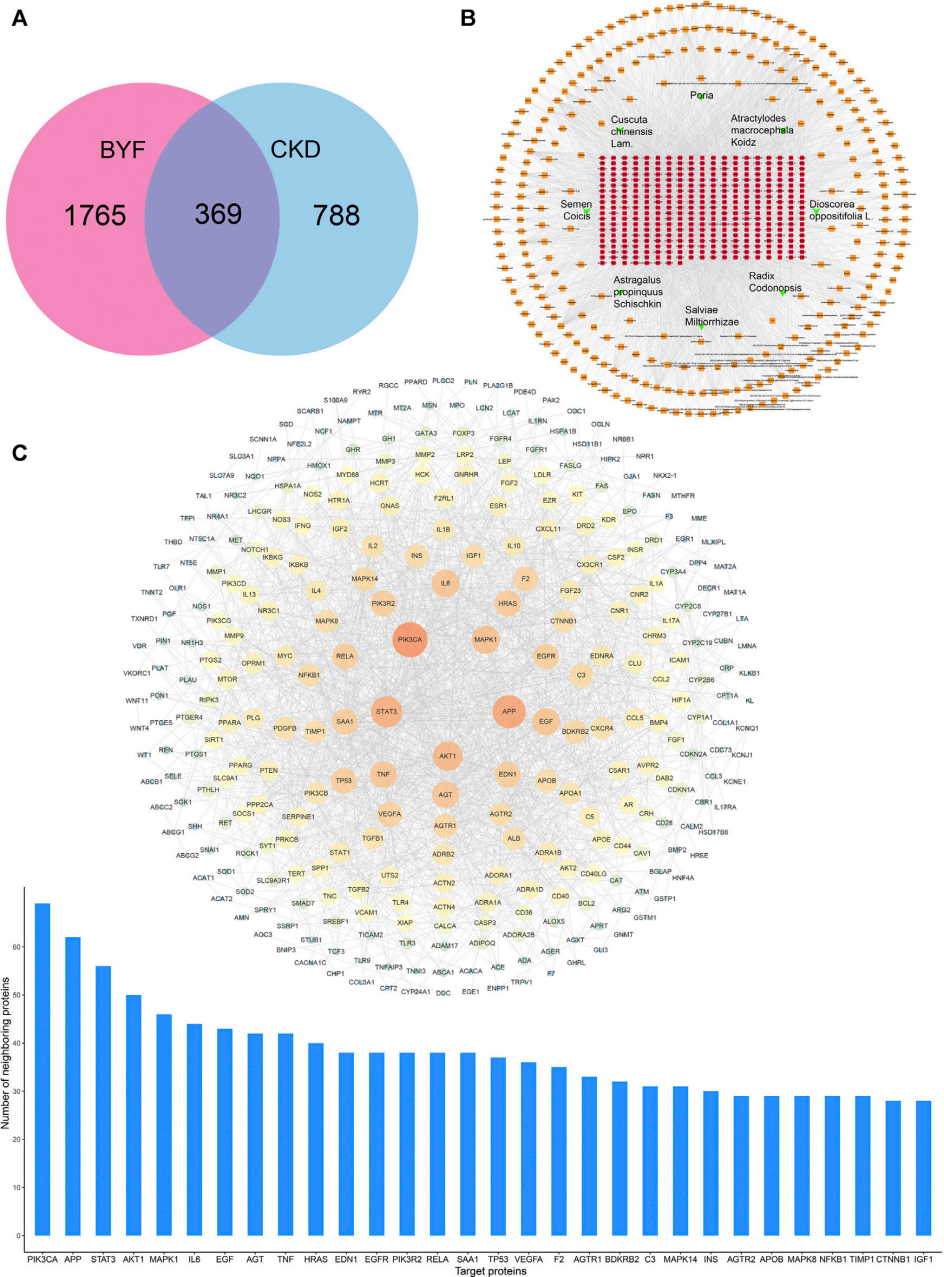
BYF network pharmacology analysis against CKD. **(A)** Venn diagram revealing BYF and CKD common target genes; **(B)** Drug-compound–target interaction network diagram; **(C)** PPI network and the proteins with the top 30° among common BYF and CKD targets.

Using TCMSP, TCMID, and Batman-TCM databases, 603 chemical compounds were screened in BYF’s eight components. Based on the DL ≥ 0.18 and OB ≥ 30%, 294 active compounds were selected acting on 2134 potential targets (Supplementary Tables S2, S3). According to OMIM, TTD, MALACARDS, and DisGeNET databases, 1,157 predicted CKD-associated targets were obtained (Supplementary Table S4). After merging active compounds and CKD targets, 369 common targets were recognized as candidates, which could be the biological basis of BYF’s effect on CKD (Figure 1A and Supplementary Table S5). Through network analysis, a drug-compound-target interaction network was established to generate the BYF active compounds for 369 candidate targets based on the 294 active compounds identified in the eight herbs (Figure 1B). From this network, we found that different compounds act on multiple targets, and vice versa. Additionally, a PPI network was established based on the 369 candidate targets by importing the candidate targets gene IDs to the STRING database (Figure 1C). The PPI network showed that there is a close interaction between those targets. The 30 core targets obtained from this PPI network showed that PI3K, AKT, IL6, TNF, NF-κb, and TLR4 were the most relevant (Supplementary Table S6).

### Functional Enrichment Analysis of BYF Candidate Targets in CKD

**FIGURE 2:**
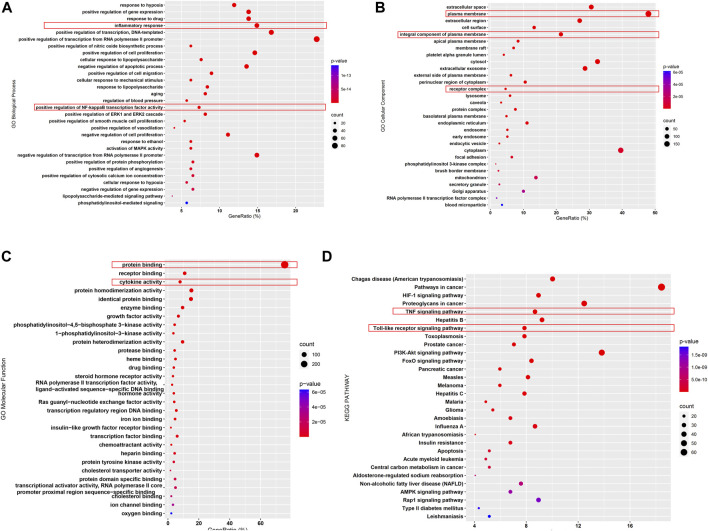
Enrichment analysis of BYF candidate targets against CKD. GO enrichment analysis revealed the related biological process **(A)**, molecular function **(B)**, and cellular component **(C)**; KEGG pathway analysis revealed the related pathways **(D)**.

GO functional enrichment analysis was annotated from three aspects: biological process, molecular function, and cellular component. To discover the BYF pharmacological mechanisms in CKD treatment, 369 candidate targets were inputted to the DAVID 6.8 database for GO enrichment analysis (Figures 2A–C). The results indicated that the following mechanisms were related to BYF against CKD: inflammatory response, NF-κB transcription factor activation, apoptotic process, plasma membranes, and protein binding. To further explore the relationship between these candidate targets and their corresponding pathways, KEGG pathway enrichment analysis was performed *via* the DAVID 6.8 database (Figure 2D). The results indicated the Toll-like receptor (TLR) signaling, TNF signaling, PI3K-AKT signaling, apoptosis, and HIF-1 signaling pathways are related to BYF mechanisms against CKD.

### BYF Improved Renal Function in an Adenine-Induced CKD Rat Model

**FIGURE 3:**
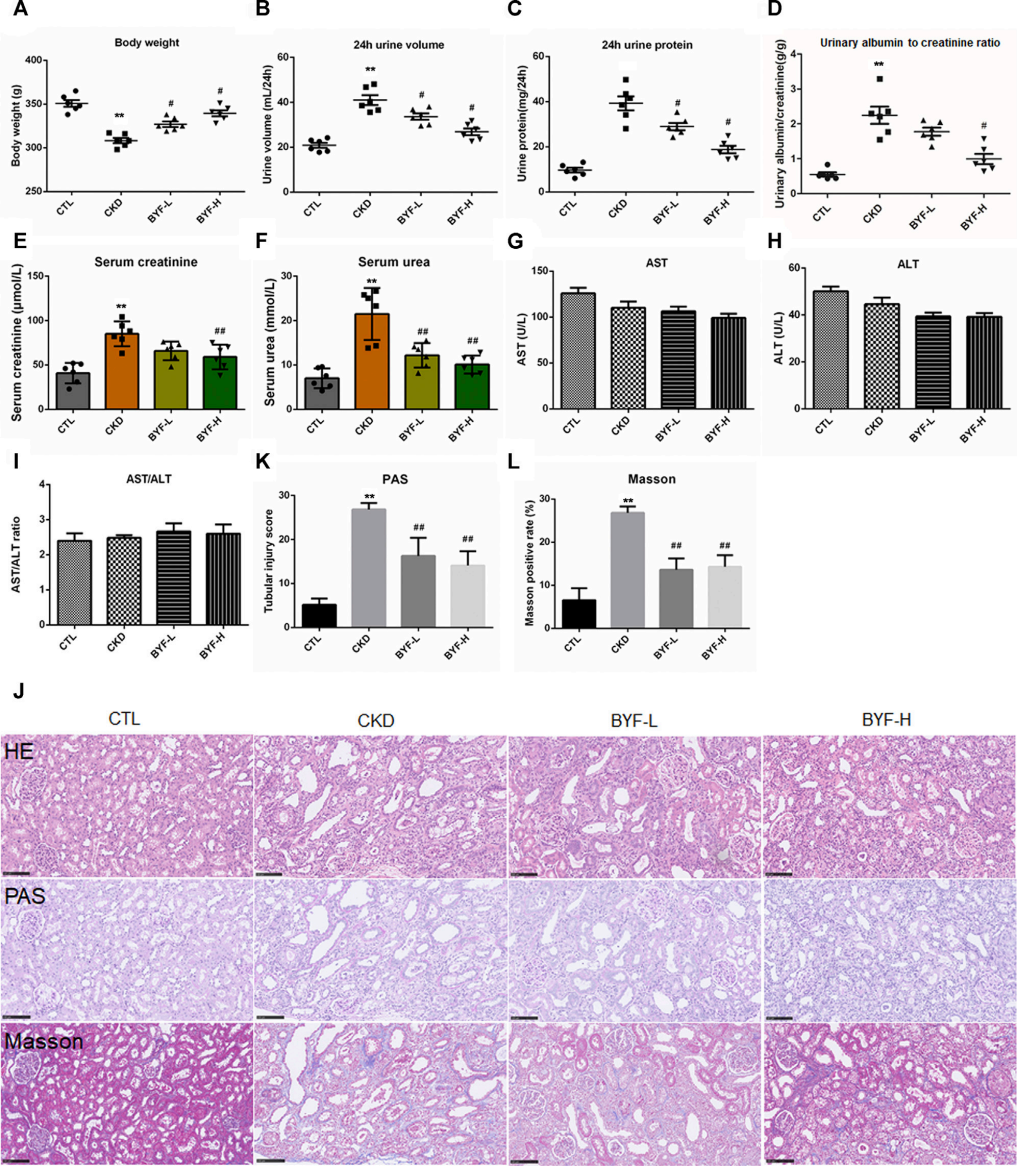
BYF improves kidney function and reduces renal pathological injury in adenine-induced CKD rats. BYF administration effectively increased body weight **(A)** and reduced urine volume **(B)**. In CKD rats, BYF reduced 24 h urinary protein excretion **(C)**, urinary albumin-to-creatinine ratio **(D)**, serum creatinine **(E)** and urea **(F)**. **(G)** Serum ALT, **(H)** AST, **(I)** and ALT/AST levels. **(J)** HE, PAS, and Masson’s trichrome staining representative micrographs in indicated groups (scale bar = 100 μm). **(K)** Chronic tubular atrophy score. **(L)** Quantitative analysis of Masson trichromatic positive rate. All values were presented as means ± SD. n = 6 rats per group. *p < 0.05 and **p < 0.01 vs. the CTL group; ^#^ *p* < 0.05 and ^##^ *p* < 0.01 vs. the CKD group.

Treatment with BYF significantly increased the body weight and decreased the urine volume in adenine-induced CKD rats (Figures 1A,B). Compared with CTL, the levels of 24 h urinary protein excretion and urine albumin-to-creatinine ratio in CKD were increased, and in BYF groups they were reduced (Figures 1C,D). The CKD group showed higher serum creatinine and urea compared to CTL, which could be restored by BYF treatment (Figures 1E,F). Moreover, the ALT and AST serum levels were not significantly different between the four groups (Figures 1G–I). This indicates that the two BYF treatment dosages that we utilized are safe. In HE staining, CKD rats showed obvious renal tubular dilation and massive inflammatory cells infiltration, and BYF treatment inhibited these changes. In PAS staining, CKD rats indicated severe loss of tubular epithelial cells, chronic tubular atrophy, and glomerulosclerosis, recovered by BYF (Figures 3J,K). Masson staining showed interstitial fibrosis in the CKD, and that BYF treatment significantly decreased collagen deposition (Figures 3J–L). Altogether, these results suggested that the CKD model was successfully established and that BYF improved kidney function and reduced structural damage in CKD rats.

### BYF Attenuated Renal Fibrosis and Inflammation in Adenine-Induced CKD Rats

**FIGURE 4:**
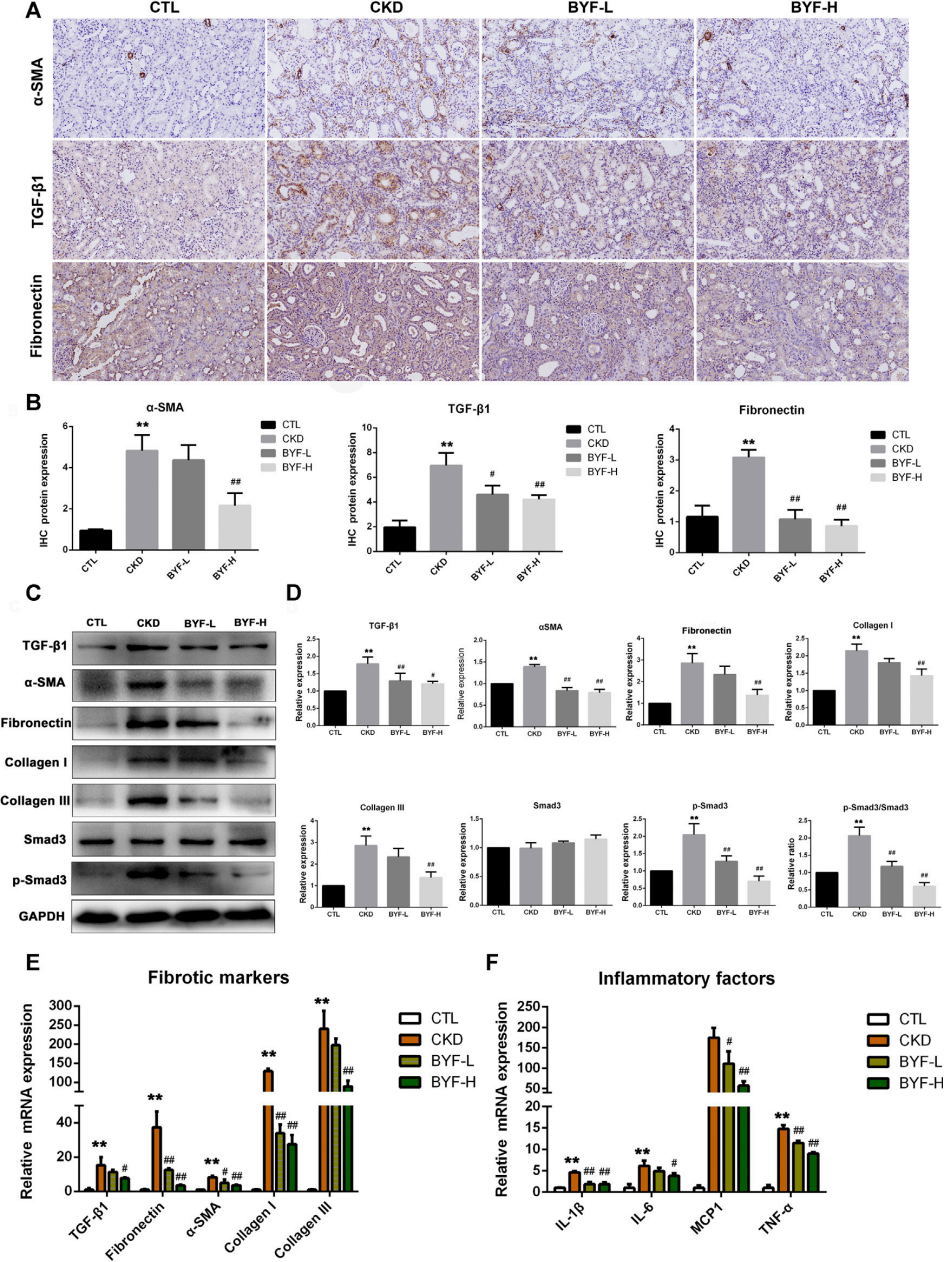
BYF reduces fibrotic and inflammatory markers expression levels in the kidney of adenine-induced CKD rats. α-SMA, TGF-β1, and Fibronectin immunohistochemical staining representative micrographs **(A)** and quantitative analysis **(B)**. TGF-β1, Fibronectin, α-SMA, Collagen I and III, and p-Smad3 WB vaαlidation **(C)** and quantitative analysis **(D)**. qPCR validation of fibrotic markers **(E)** and proinflammatory factors **(F)** in indicated groups. Scale bar = 100 μm. All values are presented as means ± SD. n = 3 rats per group. *p < 0.05 and **p < 0.01 vs. the CTL group; ^#^ *p* < 0.05 and ^##^ *p* < 0.01 vs. the CKD group.

Renal fibrosis has been long considered as the common and final manifestation of nearly all CKD progressive forms. Thus, we further examined the BYF effect on renal fibrosis by fibrotic markers. Immunohistochemical staining showed that the α-SMA, TGF-β1, and Fibronectin protein levels increased in CKD, which was decreased by BYF treatment (Figures 4A,B). Western blotting (WB) indicated that the expression levels of TGF-β1, Fibronectin, α-SMA, Collagen I and III, and p-Smad3 also increased in CKD. In contrast, BYF administration significantly reduced these proteins level (Figures 4C,D). Similarly, fibrotic markers mRNA expression increased in CKD and was reversed by BYF treatment (Figure 4E). Interestingly, BYF treatment also remarkedly downregulated proinflammatory factors mRNA level, including interleukin-1β (IL-1β), IL-6, MCP-1, and tumor necrosis factor-alpha (TNF-α) in the kidneys of CKD rats (Figure 4F). Altogether, these results suggested that BYF could inhibit the increase in TGF-β1/Smad3-mediated fibrotic markers production and reduced inflammatory cytokines release. This provided further evidence of the BYF’s beneficial effect on CKD.

### BYF Inhibited TLR4/NF-KB Signaling Pathway in The Kidneys of Adenine-Induced CKD Rats

**FIGURE 5:**
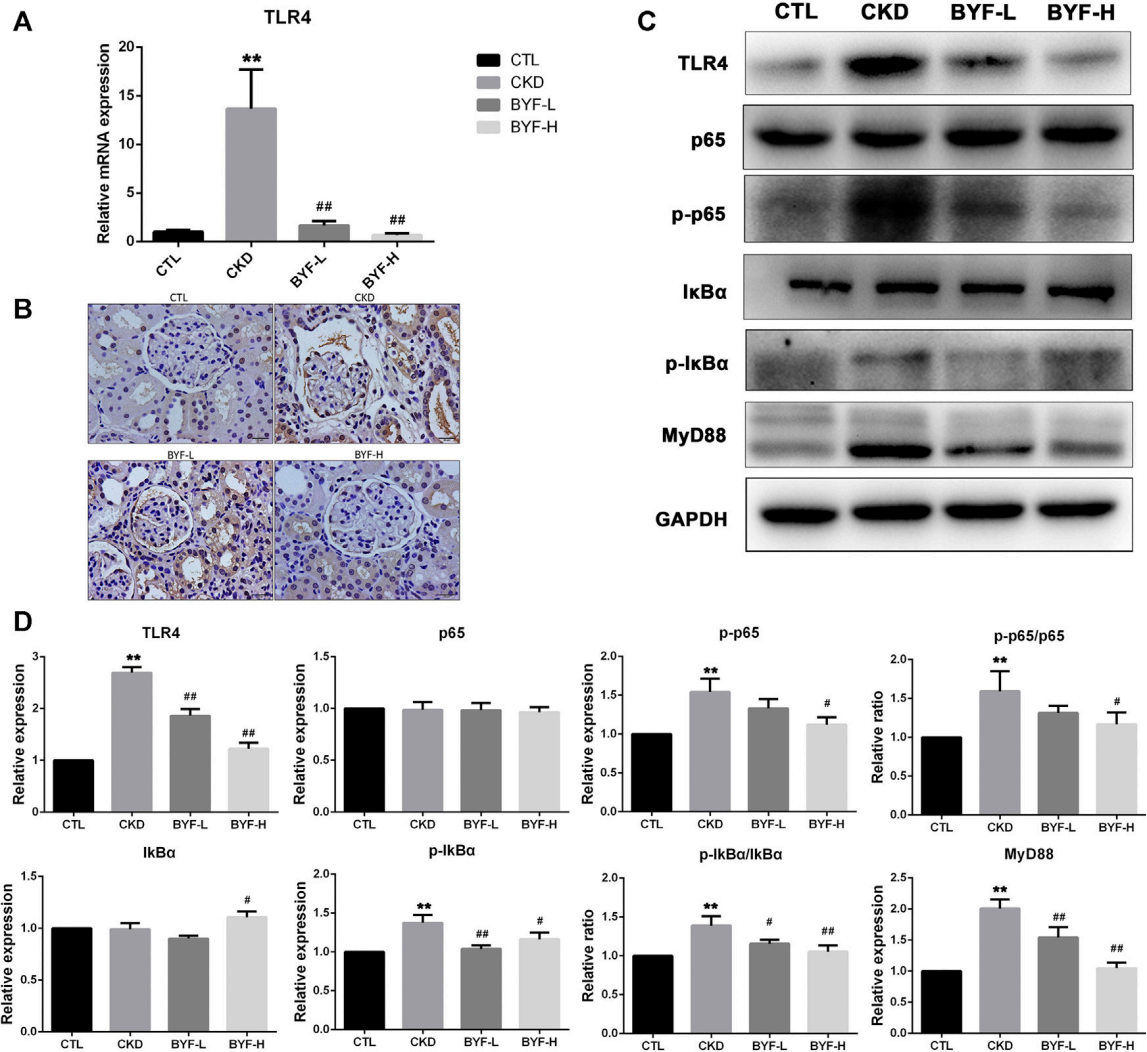
BYF suppresses TLR4/NF-κB signaling pathway in adenine-induced CKD rats. qPCR validation of TLR4 **(A)** and immunohistochemical staining of TLR4 **(B)** in indicated groups. TLR4, p65, p-p65, IKβα, p-IKβα, and MyD88 WB validation **(C)** and quantitative analysis **(D)** in indicated groups. All values are presented as means ± SD. n = 3 rats per group. *p < 0.05 and **p < 0.01 vs. the CTL group; ^#^ *p* < 0.05 and ^##^ *p* < 0.01 vs. the CKD group.

Recent studies revealed that the TLR4/NF-κB signaling pathways are closely associated with kidney fibrosis and CKD progression (Wang, et al., 2008; Liu, et al., 2015; Pérez-Ferro, et al., 2016) by augmenting TGF-β/Smads responses (Bhattacharyya, et al., 2013) and activating inflammatory cytokines (Chen, et al., 2018). Since BYF could target TLR and NF-κB pathways based on the network pharmacology analysis, the anti-fibrotic and anti-inflammatory effects of BYF on CKD were explored. The TLR4 mRNA expression and protein expression by immunohistochemical staining were significantly upregulated in CKD and downregulated by BYF (Figures 5A,B). Western blot analysis showed that the protein levels of TLR4, p-NF-κB (p-p65 and p-IKβα), and MyD88 in kidney tissues were significantly increased in CKD compared to CTL, while BYF treatment restored these proteins with the high doses effect being generally more evident (Figures 5C,D). These results indicated that BYF treatment might attenuate CKD by improving renal fibrosis and inflammation *via* TLR4/NF-κB mechanism**.**

### BYF Improved Fibrogenesis and Inflammation by Inhibiting TLR4-Mediated NFκB Signaling Pathway in TGF-β1-Induced HK2 Cells

Renal proximal tubular cells are the major sites of kidney injury and are critical in fibrosis development (Liu, et al., 2018). Based on the inhibitory effect on renal fibrosis and inflammation in adenine-induced CKD rats, TGF-β1-induced HK-2 cells were used to study the BYF protection *in vitro*. To explore an optimal BYF concentration, different concentrations (0, 32, 64, 128, 188, 256, 375, 512, 750, 1,500 mg/ml) were added to HK-2 cells for 24 h. CCK-8 assay showed that the cell viability at 32 mg/ml was optimum (Supplementary Figure S2). Based on these results, 32 mg/ml of BYF was used in the subsequent experiments.

**FIGURE 6:**
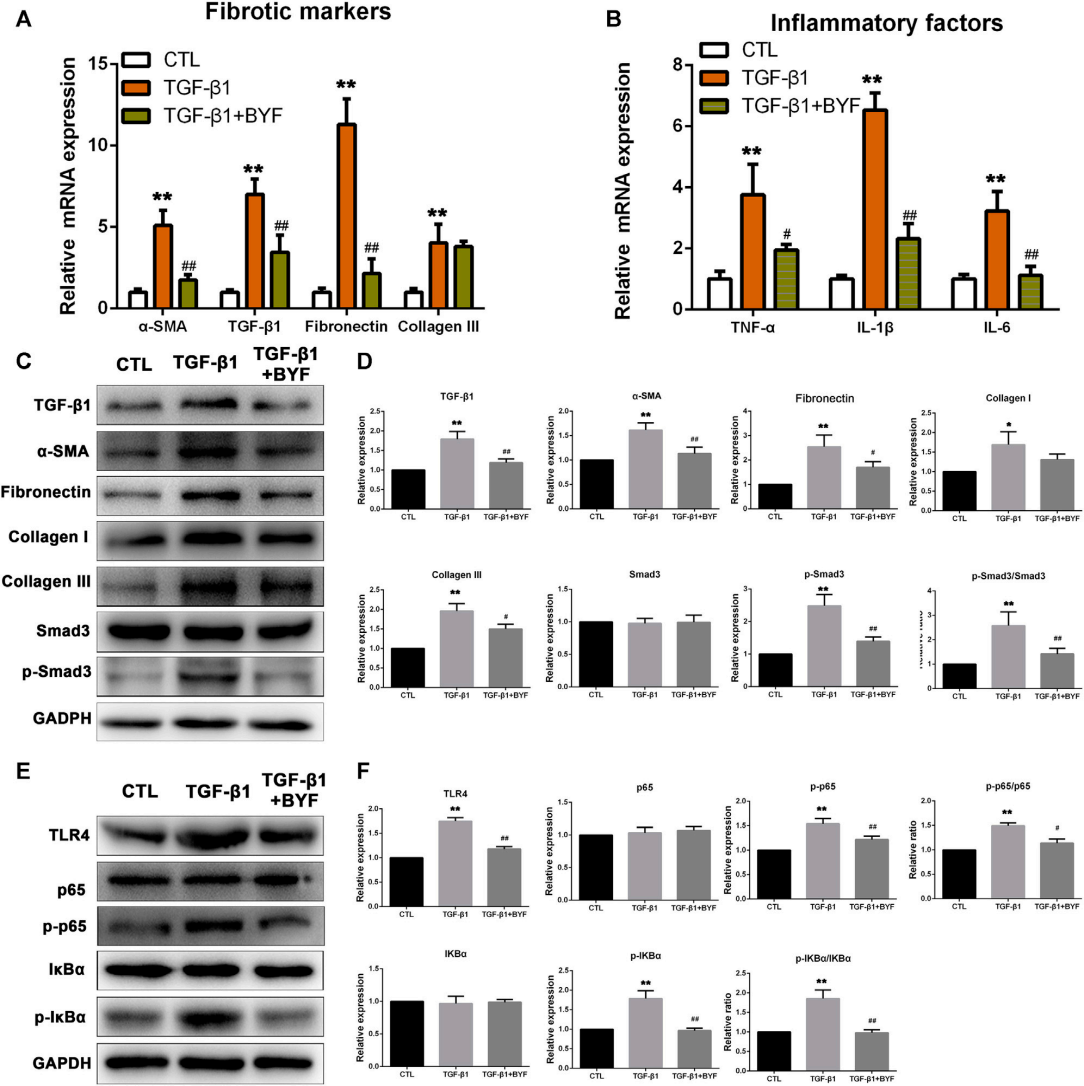
BYF treatment decreases fibrotic and inflammatory markers expression levels and inhibits TLR4/NF-κB signaling pathway in the TGF-β1-induced HK2 cells. qPCR validation of fibrotic markers **(A)** and inflammatory factors **(B)** between groups. Fibrotic markers WB validation **(C)** and quantitative analysis **(D)** in indicated groups. TLR4/NF-KB signaling main proteins WB validation **(E)** and quantitative analysis **(F)** in indicated groups. All values are presented as means ± SD. n = 3 independent experiments for each indicator. *p < 0.05 and **p < 0.01 vs. the CTL group; ^#^ *p* < 0.05 and ^##^ *p* < 0.01 vs. the CKD group.

To evaluate the anti-fibrotic and anti-inflammatory effects of BYF *in vitro*, the fibrotic and inflammatory markers expression level was detected in TGF-β1-induced HK-2 cells. Results showed that BYF markedly reduced the elevated α-SMA, Fibronectin, and TGF-β1, as well as TNF-α, IL-1β, and IL-6 mRNA levels in TGF-β1-induced HK-2 cells (Figure 6A,B). Likewise, TGF-β1, α-SMA, Fibronectin, Collagen III, and p-Smad3 protein expression levels reduced after BYF treatment (Figures 6C,D). Next, we investigated the mechanisms whereby BYF inhibits renal fibrosis and inflammation in TGF-β1-induced HK-2 cells. Cells treated with TGF-β1 increased the TLR4, p-p65, and p-IKβα protein levels, while BYF treatment reduced it (Figures 6E,F). These results suggested that BYF likely inhibited fibrogenesis and inflammation *in vitro* by TLR4/NF-KB pathway suppression.

**FIGURE 7:**
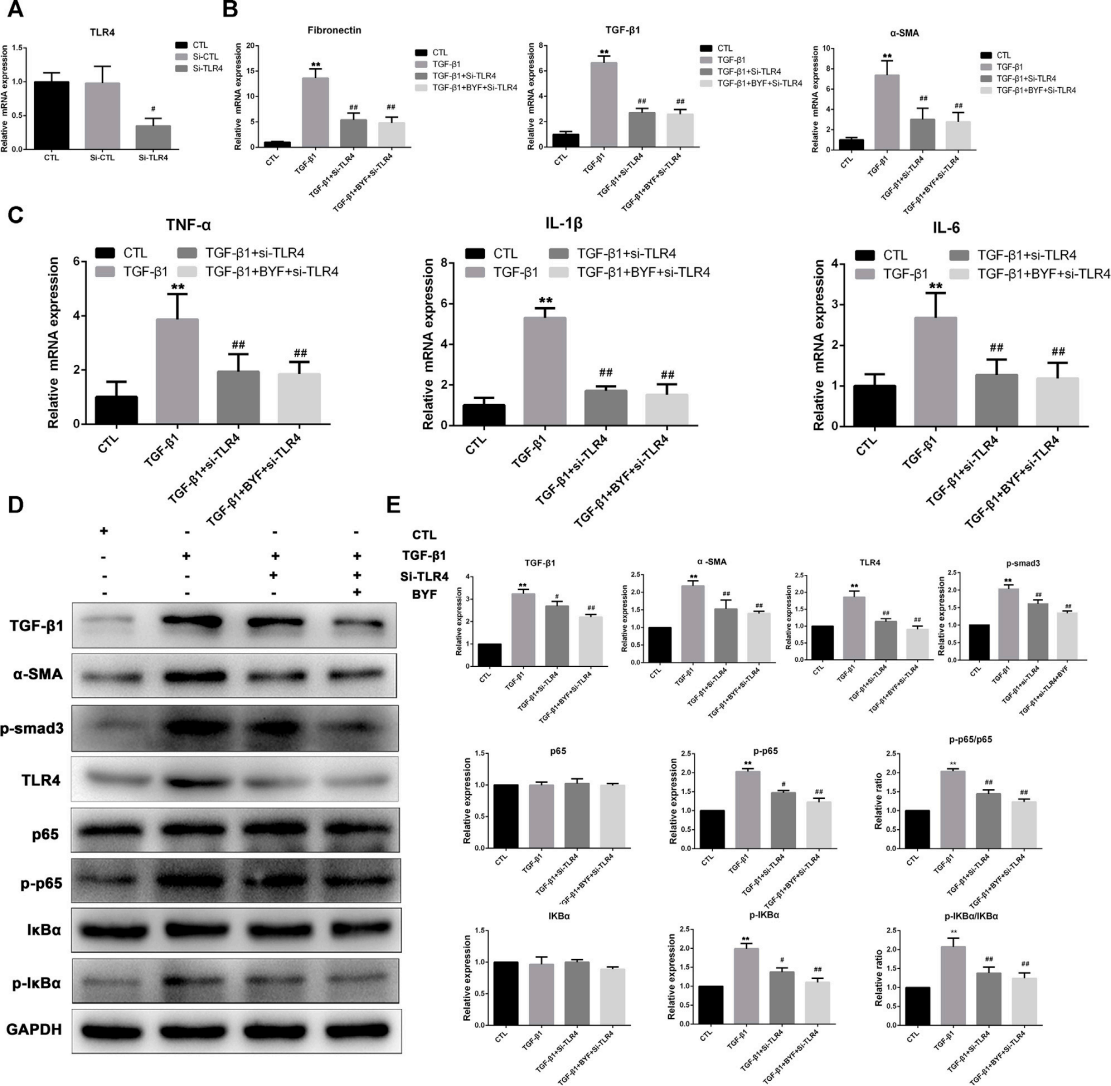
Silencing TLR4 significantly suppresses BYF inhibitory effect on TGF-β1-induced fibrogenesis and inflammation and NF-κB pathway activation in HK2 cells. **(A)** TLR4 qPCR validation in HK-2 cells transfected with TLR4 siRNA. **(B)** Fibrotic markers qPCR validation between groups. **(C)** Inflammatory factors qPCR validation between groups. TLR4/NF-κB signaling rescue assay WB validation **(D)** and quantitative analysis **(E)** in indicated groups. All values are presented as means ± SD. n = 3 independent experiments for each indicator. *p < 0.05 and **p < 0.01 vs. the CTL group; ^#^ *p* < 0.05 and ^##^ *p* < 0.01 vs. the CKD group.

To better understand TRL4/NF-κb signaling functional role in TRL4-mediated renal fibrosis and inflammation, we knocked down TRL4 in HK2 cells by siRNA. TLR4 mRNA expression was significantly downregulated (Figure 7A). The expression levels of fibrotic and inflammatory markers showed that BYF treatment could not markedly decrease the fibronectin, TGF-β1, and α-SMA mRNA levels, as well as TNF-α, IL-1β, and IL-6 in TGF-β1-induced HK-2 cells silenced with TLR4 siRNA (Figures 7B,C). Next, WB showed that silencing TRL4 markedly suppressed the TGF-β1 activation effect on the NF-κb pathway in HK2 cells with reduced p-p65, and p-IKβα expression levels, compared with the TGF-β1 group. Furthermore, BYF did not significantly reduce the levels when TLR4 was silenced (Figures 7D,E). Altogether, these results demonstrated that the BYF might have a protective effect *via* the TLR4-mediated NF-κB mechanism to reduce CKD renal fibrosis and inflammation.

## Discussion

**FIGURE 8:**
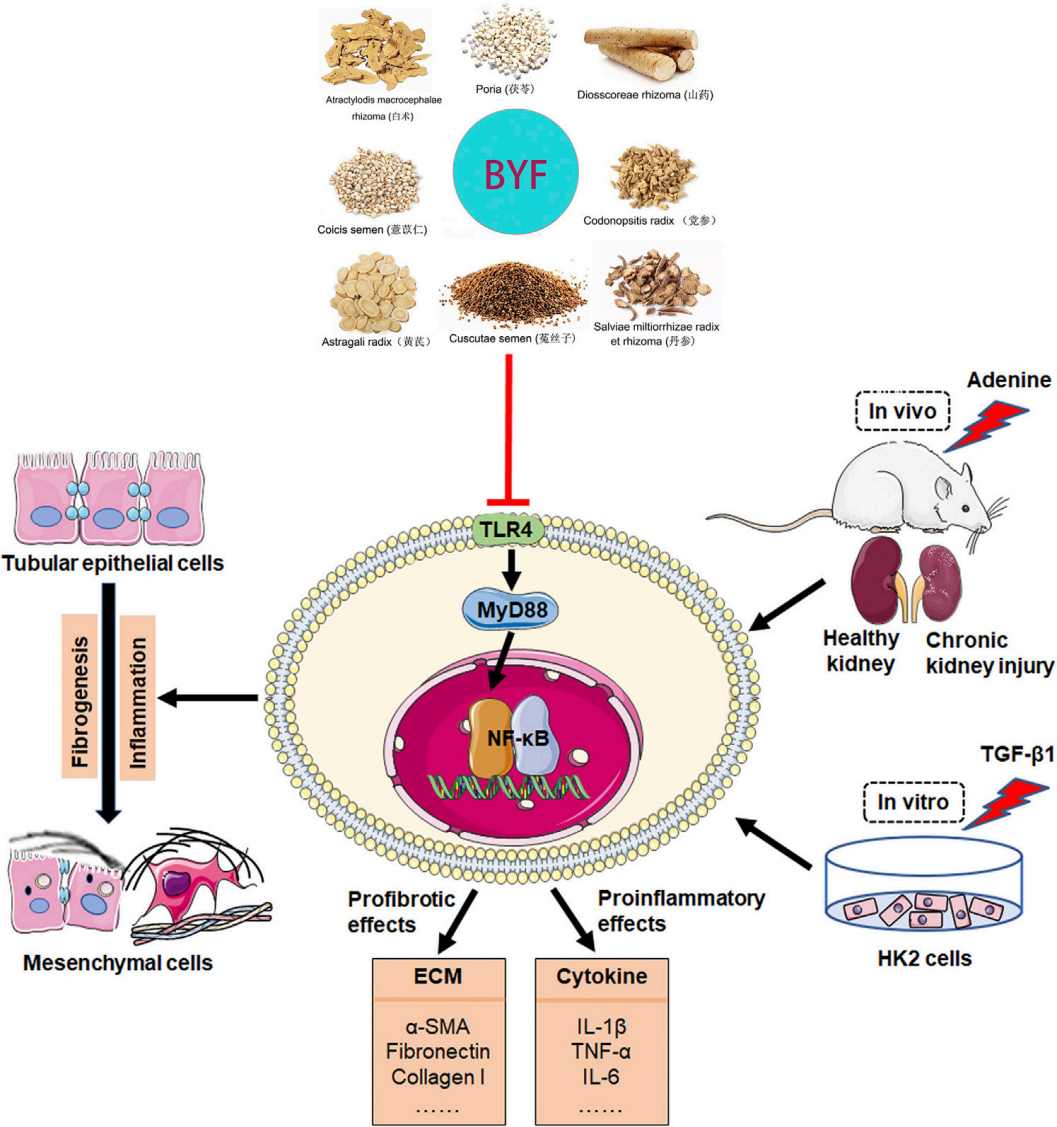
BYF regulation on the TLR4/NF-κb signaling pathway in CKD.

Although our previous clinical study revealed that BYF have protective effects on delaying kidney function progression among advanced CKD patients (Mao, et al., 2020), its pharmacological mechanisms remain ambiguous. In this study, we investigated the BYF effect on CKD *in vivo* and *in vitro.* We explored its potential mechanisms combining network pharmacology, histopathology, and molecular biology. Network pharmacology-based analysis predicted that BYF protected against CKD through the TLR/NF-κB signaling pathway and the inflammatory and fibrotic response triggered by this pathway. Experimental validation indicated that BYF effectively inhibited the fibrosis and inflammatory response in adenine-induced CKD rats and TGF-β1-induced HK-2 cells. Also, these results showed that BYF alleviated renal fibrosis and inflammation *via* TLR4/NF-κB signaling pathway modulation. The putative anti-fibrotic and anti-inflammatory BYF mechanism in CKD is shown in Figure 8.

BYF is composed of eight Chinese herbs and has complex bioactive compounds. Therefore, it is difficult to clearfy its molecular mechanism through traditional pharmacological techniques. Network pharmacology-based analysis integrates a series of disciplines and techniques, including genomics, proteomics, and systems biology (Kim, et al., 2019). Thus, network pharmacology provides an effective method to clarify the multifaceted biological phenomenon mechanism of such complex compounds in Chinese herbal formulations. To explore the BYF molecular mechanism, we selected 369 common BYF and CKD targets, constructed a drug-compound-target network, and executed GO and KEGG enrichment analysis. Results suggested that TLR and NF-κB signaling pathway plays an important role in BYF pharmacological mechanism during CKD treatment. Compounds associated with BYF may directly act on TLR4/NF-κB signaling pathway and interfere with the downstream TGF-β1/Smad3 signaling pathway and inflammatory response, resulting in the inhibition of profibrotic factors (α-SMA, Fibronectin, Collagen I and III) as well as the release of proinflammation cytokines (TNF-α, IL-1β, IL-8). Interestingly, the network pharmacology results showed that BYF may also interfere with the downstream targets, like PI3K, AKT, and TNF (except for the main molecules in the TLR4/NF-κB pathway), with or without TLR2/3 interference. These findings indicated that BYF may interfere with CKD by multiple pathways through multiple compounds from different Chinese herbs.

Many animal models have been developed to study CKD pathogenesis and treatment in humans. However, most models do not mimic CKD complexity in humans, and the adenine diet or gavage model in rodents is an exception (Yokozawa, et al., 1986; Diwan, et al., 2018). Intra-gastric gavage of 200 mg/kg adenine in rats for 4 weeks has been well-accepted as a model to study kidney damage since this intervention mimick most of the functional and structural changes observed in human CKD (Chen, et al., 2017; Yang H, et al., 2018). We observed high levels of proteinuria, serum creatinine and urea, as well as tubular atrophy, inflammatory cells infiltration, and collagen synthesis during the 4 weeks in the adenine-induced CKD rats. Furthermore, BYF treatment partially recovered the kidney dysfunction and histopathological injury compared to the adenine-induced CKD group. These results suggested that a successful CKD rat model induced by adenine gavage was established.

Inflammation and fibrosis are two CKD pathological features. It has been proven that TGF-β1, α-SMA, and extracellular matrix (ECM) proteins such as fibronectin, and collagen I and III are master markers in kidney fibrosis development (Zeisberg and Neilson, 2010; Liu, 2011). Our results indicated that BYF markedly inhibited these fibrotic markers expression levels in adenine-induced CKD rat and TGF-β1-induced HK-2 cells, suggesting its anti-fibrotic effect in the kidney. Also, we found that BYF significantly decreased the proinflammatory factors mRNA levels, including IL-1β, IL-6, MCP-1, and TNF-α, *in vivo* and *in vitro*, indicating its anti-inflammatory effect in the kidney. Besides, TGF-β/Smads signaling prominent activation was observed (with increased TGF-β1 and p-Smad3 levels), which was reversed by BYF treatment. These results demonstrated that BYF protected against CKD through anti-fibrotic and anti-inflammatory effects.

It is well known that the TLR signaling pathway is one of the most crucial pathways in the host immune response in an infected environment, responding to different microorganisms and endogenous ligands (Mollen, et al., 2006). TLR4, an important member of the Toll-like family, is located in the cell membrane and cytoplasm and is crucial in the kidney fibrosis process (Bhattacharyya, et al., 2013; Pérez-Ferro, et al., 2016). NF-κB, an important transcription activator, modulates and regulates inflammatory mediators, and induces cytokines production (Mitchell, et al., 2016). It was reported that TLR4 enhances the downstream activation of the NF-κB pathway, which ultimately results in the inflammation reaction (Ciesielska, et al., 2021). In this study, we found that the TLR4, p-p65, p-IKβα, and MyD88 proteins levels were significantly increased in adenine-induced CKD rats, which markedly decreased after BYF treatment. These results suggested that BYF treatment may partially heal CKD by renal inflammation and fibrosis reduction *via* TLR4/NF-κB suppression mechanism.

Many kidney cell types (e.g., tubular, myofibroblasts, endothelial, and inflammatory) are involved in the development and progression of renal fibrogenesis and inflammation under pathological conditions (Liu, 2011). However, emerging evidence indicated that proximal tubular epithelial cells are the major injury sites and are critical in injury repair and fibrosis development (Yang, et al., 2010; Kang, et al., 2015; Liu, et al., 2018). TGF-β1 is one of the most powerful profibrotic cytokines and is vital in renal inflammation and fibrosis by downstream Smad3 signaling activation (Sutariya, et al., 2016; Gu, et al., 2020). It was also demonstrated that TGF-β signaling could be activated by TLR4 in a hepatic fibrogenesis mice model (Seki, et al., 2007). Therefore, we examined the BYF protective effects and TLR4/NF-κ b mechanism on TGF-β1-induced HK2 cells. We found that BYF markedly decreased fibrotic and inflammatory markers expression, and inhibited the protein expression of main molecules in the TLR4/NF-KB signaling pathway in TGF-β1-induced HK-2 cells. These results were consistent with previous research results obtained from adenine-induced CKD rat models. Then, we showed that the BYF anti-fibrotic and anti-inflammatory inhibition effects on the NF-κB pathway were diminished when TLR4 was silenced with siRNA in TGF-β1-induced HK-2 cells. Collectively, these findings demonstrated that the TLR4/NF-κB signaling suppression was an important anti-fibrotic and anti-inflammatory mechanism by which BYF partially healed CKD.

## Conclusion

Our results indicated that BYF could significantly reduce renal fibrosis and inflammation by TLR4/NF-κB signaling pathway inhibition. Although these preliminary findings could not fully explain the underlying mechanism of the BYF protective effect, they provided further support for clinical trials aiming to assess the BYF effects against CKD progression. However, whether BYF has a beneficial role in any non-adenine-induced CKD rat model needs to be further elucidated. Moreover, due to the TCM complex composition, new technologies are required to investigate the material basis and underlying mechanisms of BYF on CKD.

## Data Availability

The original contributions presented in the study are included in the article/Supplementary Material, further inquiries can be directed to the corresponding authors.

## Supplementary Material

Supplementary Figure S1 Graphical Abstract Study design: workflow used for the study.

Supplementary Figure S2 BYF dose-dependent effect on cell viability by the CCK8 assay after 24 h.

## References

[B1] Amberger J. S. Bocchini C. A. Schiettecatte F. Scott A. F. Hamosh A. (2015). OMIM.org: Online Mendelian Inheritance in Man (OMIM®), an Online Catalog of Human Genes and Genetic Disorders. Nucleic Acids Res. 43, D789–D798. 10.1093/nar/gku1205 25428349 PMC4383985

[B2] Bello A. K. Hemmelgarn B. Lloyd A. James M. T. Manns B. J. Klarenbach S. (2011). Associations Among Estimated Glomerular Filtration Rate, Proteinuria, and Adverse Cardiovascular Outcomes. Clin. J. Am. Soc. Nephrol. 6, 1418–1426. 10.2215/CJN.09741110 21527648 PMC3109940

[B3] Bhattacharyya S. Kelley K. Melichian D. S. Tamaki Z. Fang F. Su Y. (2013). Toll-like Receptor 4 Signaling Augments Transforming Growth Factor-β Responses: a Novel Mechanism for Maintaining and Amplifying Fibrosis in Scleroderma. Am. J. Pathol. 182, 192–205. 10.1016/j.ajpath.2012.09.007 23141927 PMC3538029

[B4] Breyer M. D. Susztak K. (2016). Developing Treatments for Chronic Kidney Disease in the 21st Century. Semin. Nephrol. 36, 436–447. 10.1016/j.semnephrol.2016.08.001 27987541 PMC5423404

[B5] Chen C. Y. Kao C. L. Liu C. M. (2018). The Cancer Prevention, Anti-inflammatory and Anti-oxidation of Bioactive Phytochemicals Targeting the TLR4 Signaling Pathway. Int. J. Mol. Sci. 19, 2729. 10.3390/ijms19092729 PMC6164406 30213077

[B6] Chen D. Q. Chen H. Chen L. Vaziri N. D. Wang M. Li X. R. (2017). The Link between Phenotype and Fatty Acid Metabolism in Advanced Chronic Kidney Disease. Nephrol. Dial. Transpl. 32, 1154–1166. official publication of the European Dialysis and Transplant Association - European Renal Association. 10.1093/ndt/gfw415 28339984

[B7] Ciesielska A. Matyjek M. Kwiatkowska K. (2021). TLR4 and CD14 Trafficking and its Influence on LPS-Induced Pro-inflammatory Signaling. Cell Mol Life Sci 78, 1233–1261. 10.1007/s00018-020-03656-y 33057840 PMC7904555

[B8] Diwan V. Brown L. Gobe G. C. (2018). Adenine-induced Chronic Kidney Disease in Rats. Nephrology (Carlton) 23, 5–11. 10.1111/nep.13180 29030945

[B9] Feng X. Fang S. N. Gao Y. X. Liu J. P. Chen W. (2018). Current Research Situation of Nephrotoxicity of Chinese Herbal Medicine. Zhongguo Zhong Yao Za Zhi 43, 417–424. 10.19540/j.cnki.cjcmm.2018.0009 29600603

[B10] Gu Y. Y. Liu X. S. Huang X. R. Yu X. Q. Lan H. Y. (2020). Diverse Role of TGF-β in Kidney Disease. Front Cel Dev Biol 8, 123. 10.3389/fcell.2020.00123 PMC7093020 32258028

[B11] Jha V. Garcia-Garcia G. Iseki K. Li Z. Naicker S. Plattner B. (2013). Chronic Kidney Disease: Global Dimension and Perspectives. Lancet 382, 260–272. 10.1016/S0140-6736(13)60687-X 23727169

[B12] Kang H. M. Ahn S. H. Choi P. Ko Y. A. Han S. H. Chinga F. (2015). Defective Fatty Acid Oxidation in Renal Tubular Epithelial Cells Has a Key Role in Kidney Fibrosis Development. Nat. Med. 21, 37–46. 10.1038/nm.3762 25419705 PMC4444078

[B13] Kim S. K. Lee S. Lee M. K. Lee S. (2019). A Systems Pharmacology Approach to Investigate the Mechanism of Oryeong-San Formula for the Treatment of Hypertension. J. Ethnopharmacol 244, 112129. 10.1016/j.jep.2019.112129 31376514

[B14] Lameire N. Jager K. Van Biesen W. I. M. de Bacquer D. Vanholder R. (2005). Chronic Kidney Disease: A European Perspective. Kidney Int. 68 (Suppl. ment), S30–S38. 10.1111/j.1523-1755.2005.09907.x 16336574

[B15] Li P. Lin H. Ni Z. Zhan Y. He Y. Yang H. (2020). Efficacy and Safety of Abelmoschus Manihot for IgA Nephropathy: A Multicenter Randomized Clinical Trial. Phytomedicine 76, 153231. 10.1016/j.phymed.2020.153231 32535481

[B16] Li X. Wang H. (2005). Chinese Herbal Medicine in the Treatment of Chronic Kidney Disease. Adv. Chronic Kidney Dis. 12, 276–281. 10.1016/j.ackd.2005.03.007 16010642

[B17] Lin M. Y. Chiu Y. W. Chang J. S. Lin H. L. Lee C. T. Chiu G. F. (2015). Association of Prescribed Chinese Herbal Medicine Use with Risk of End-Stage Renal Disease in Patients with Chronic Kidney Disease. Kidney Int. 88, 1365–1373. 10.1038/ki.2015.226 26244923

[B18] Liu B. C. Tang T. T. Lv L. L. Lan H. Y. (2018). Renal Tubule Injury: a Driving Force toward Chronic Kidney Disease. Kidney Int. 93, 568–579. 10.1016/j.kint.2017.09.033 29361307

[B19] Liu Y. (2011). Cellular and Molecular Mechanisms of Renal Fibrosis. Nat. Rev. Nephrolnephrology 7, 684–696. 10.1038/nrneph.2011.149 PMC4520424 22009250

[B20] Liu Z. Kan Y. H. Wei Y. D. Li X. J. Yang F. Hou Y. (2015). Decreased Number of CD14+TLR4+ Monocytes and Their Impaired Cytokine Responses to Lipopolysaccharide in Patients with Chronic Kidney Disease. J. Huazhong Univ. Sci. Technolog Med. Sci. 35, 206–211. Medical sciences = Hua zhong ke ji da xue xue bao. Yi xue Ying De wen ban = Huazhong keji daxue xuebao. Yixue Yingdewen ban. 10.1007/s11596-015-1412-7 25877353

[B21] Mao W. Yang N. Zhang L. Li C. Wu Y. Ouyang W. (2020). Bupi Yishen Formula versus Losartan for Non-diabetic Stage 4 Chronic Kidney Disease: A Randomized Controlled Trial. Front. Pharmacol. 11, 627185. 10.3389/fphar.2020.627185 33708125 PMC7941267

[B22] Mitchell S. Vargas J. Hoffmann A. (2016). Signaling via the NFκB System. Wiley Interdiscip. Rev. Syst. Biol. Med. 8, 227–241. 10.1002/wsbm.1331 26990581 PMC8363188

[B23] Mollen K. P. Anand R. J. Tsung A. Prince J. M. Levy R. M. Billiar T. R. (2006). Emerging Paradigm: Toll-like Receptor 4-sentinel for the Detection of Tissue Damage. Shock 26, 430–437. 10.1097/01.shk.0000228797.41044.08 17047512

[B24] Omer Mohamed H. A. Osman O. M. Ali H. H. Asiri M. N. Hassan A. A. Almangah I. M. (2020). A Complicated Chinese Herbal Medicine Nephrotoxicity. Saudi J. Kidney Dis. Transplsaudi Arabia 31, 533–536. an official publication of the Saudi Center for Organ Transplantation. 10.4103/1319-2442.284032 32394930

[B25] Palmer S. C. Mavridis D. Navarese E. Craig J. C. Tonelli M. Salanti G. (2015). Comparative Efficacy and Safety of Blood Pressure-Lowering Agents in Adults with Diabetes and Kidney Disease: a Network Meta-Analysis. Lancet 385, 2047–2056. 10.1016/S0140-6736(14)62459-4 26009228

[B26] Pérez-Ferro M. Serrano Del Castillo C. Sánchez-Pernaute O. (2016). Cell Membrane-Bound TLR2 and TLR4: Potential Predictors of Active Systemic Lupus Erythematosus and Lupus Nephritis. J. Rheumatol. 43, 1444–1445. 10.3899/jrheum.151386 27371649

[B27] Piñero J. Bravo À. Queralt-Rosinach N. Gutiérrez-Sacristán A. Deu-Pons J. Centeno E. (2017). DisGeNET: a Comprehensive Platform Integrating Information on Human Disease-Associated Genes and Variants. Nucleic Acids Res. 45, D833–833D839. 10.1093/nar/gkw943 27924018 PMC5210640

[B28] Rappaport N. Twik M. Plaschkes I. Nudel R. Iny Stein T. Levitt J. (2017). MalaCards: an Amalgamated Human Disease Compendium with Diverse Clinical and Genetic Annotation and Structured Search. Nucleic Acids Res. 45, D877–877D887. 10.1093/nar/gkw1012 27899610 PMC5210521

[B29] Ru J. Li P. Wang J. Zhou W. Li B. Huang C. (2014). TCMSP: a Database of Systems Pharmacology for Drug Discovery from Herbal Medicines. J. Cheminform 6, 13. 10.1186/1758-2946-6-13 24735618 PMC4001360

[B30] Seki E. De Minicis S. Osterreicher C. H. Kluwe J. Osawa Y. Brenner D. A. (2007). TLR4 Enhances TGF-Beta Signaling and Hepatic Fibrosis. Nat. Med. 13, 1324–1332. 10.1038/nm1663 17952090

[B31] Stelzer G. Dalah I. Stein T. I. Satanower Y. Rosen N. Nativ N. (2011). In-silico Human Genomics with GeneCards. Hum. Genomics 5, 709–717. 10.1186/1479-7364-5-6-709 22155609 PMC3525253

[B32] Sutariya B. Jhonsa D. Saraf M. N. (2016). TGF-β: the Connecting Link between Nephropathy and Fibrosis. Immunopharmacol Immunotoxicol 38, 39–49. 10.3109/08923973.2015.1127382 26849902

[B33] Thakur R. Sharma A. Lingaraju M. C. Begum J. Kumar D. Mathesh K. (2018). Ameliorative Effect of Ursolic Acid on Renal Fibrosis in Adenine-Induced Chronic Kidney Disease in Rats. Biomed. Pharmacother. 101, 972–980. 10.1016/j.biopha.2018.02.143 29635907

[B34] von Mering C. Jensen L. J. Snel B. Hooper S. D. Krupp M. Foglierini M. (2005). STRING: Known and Predicted Protein-Protein Associations, Integrated and Transferred across Organisms. Nucleic Acids Res. 33, D433–D437. 10.1093/nar/gki005 15608232 PMC539959

[B35] Wang H. Jiang X. M. Xu J. H. Xu J. Tong J. X. Wang Y. W. (2008). The Profile of Gene Expression and Role of Nuclear Factor Kappa B on Glomerular Injury in Rats with Thy-1 Nephritis. Clin. Exp. Immunol. 152, 559–567. 10.1111/j.1365-2249.2008.03654.x 18422731 PMC2453208

[B36] Wang Y. Zheng C. Huang C. Li Y. Chen X. Wu Z. (2015). Systems Pharmacology Dissecting Holistic Medicine for Treatment of Complex Diseases: An Example Using Cardiocerebrovascular Diseases Treated by TCM. *Evidence-Based Complementary and Alternative Medicine* . eCAM 2015, 980190. 10.1155/2015/980190 26101539 PMC4460250

[B37] Wojcikowski K. Johnson D. W. Gobe G. (2006). Herbs or Natural Substances as Complementary Therapies for Chronic Kidney Disease: Ideas for Future Studies. J. Lab. Clin. Med. 147, 160–166. 10.1016/j.lab.2005.11.011 16581343

[B38] Wu Y. Li C. Zhang L. Zou C. Xu P. Wen Z. (2021). Effectiveness of Chinese Herbal Medicine Combined with Western Medicine on Deferring Dialysis Initiation for Nondialysis Chronic Kidney Disease Stage 5 Patients: a Multicenter Prospective Nonrandomized Controlled Study. Ann. Transl Med. 9, 490. 10.21037/atm-21-871 33850887 PMC8039672

[B39] Wu Y. Zhang F. Yang K. Fang S. Bu D. Li H. (2019). SymMap: an Integrative Database of Traditional Chinese Medicine Enhanced by Symptom Mapping. Nucleic Acids Res. 47, D1110–1110D1117. 10.1093/nar/gky1021 30380087 PMC6323958

[B40] Yang B. Xie Y. Guo M. Rosner M. H. Yang H. Ronco C. (2018). Nephrotoxicity and Chinese Herbal Medicine. Clin. J. Am. Soc. Nephrol. 13, 1605–1611. 10.2215/CJN.11571017 29615394 PMC6218812

[B41] Yang H. Song Y. Liang Y. N. Li R. (2018). Quercetin Treatment Improves Renal Function and Protects the Kidney in a Rat Model of Adenine-Induced Chronic Kidney Disease. Med. Sci. Monit. 24, 4760–4766. 10.12659/MSM.909259 29987270 PMC6069490

[B42] Yang L. Besschetnova T. Y. Brooks C. R. Shah J. V. Bonventre J. V. (2010). Epithelial Cell Cycle Arrest in G2/M Mediates Kidney Fibrosis after Injury. Nat. Med. 16, 535–143. 1p following 143. 10.1038/nm.2144 20436483 PMC3928013

[B43] Yokozawa T. Zheng P. D. Oura H. Koizumi F. (1986). Animal Model of Adenine-Induced Chronic Renal Failure in Rats. Nephron 44, 230–234. 10.1159/000183992 3785486

[B44] Zeisberg M. Neilson E. G. (2010). Mechanisms of Tubulointerstitial Fibrosis. J. Am. Soc. Nephrol. 21, 1819–1834. 10.1681/ASN.2010080793 20864689

[B45] Zhang J. Xu W. Wang P. Huang J. Bai J. Q. Huang Z. H. (2018). Chemical Analysis and Multi-Component Determination in Chinese Medicine Preparation Bupi Yishen Formula Using Ultra-high Performance Liquid Chromatography with Linear Ion Trap-Orbitrap Mass Spectrometry and Triple-Quadrupole Tandem Mass Spectrometry. Front. Pharmacol. 9, 568. 10.3389/fphar.2018.00568 29937729 PMC6002530

[B46] Zhang L. Li P. Xing C. Y. Zhao J. Y. He Y. N. Wang J. Q. (2014). Efficacy and Safety of Abelmoschus Manihot for Primary Glomerular Disease: a Prospective, Multicenter Randomized Controlled Clinical Trial. Am. J. Kidney Dis. 64, 57–65. 10.1053/j.ajkd.2014.01.431 24631042

[B47] Zhang L. Wang F. Wang L. Wang W. Liu B. Liu J. (2012). Prevalence of Chronic Kidney Disease in China: a Cross-Sectional Survey. Lancet 379, 815–822. 10.1016/S0140-6736(12)60033-6 22386035

[B48] Zhong Y. Deng Y. Chen Y. Chuang P. Y. Cijiang He J. (2013). Therapeutic Use of Traditional Chinese Herbal Medications for Chronic Kidney Diseases. Kidney Int. 84, 1108–1118. 10.1038/ki.2013.276 23868014 PMC3812398

