# Supplementary material for: Uncovering Bupi Yishen Formula Pharmacological Mechanisms Against Chronic Kidney Disease by Network Pharmacology and Experimental Validation
Source: Front Pharmacol. 2021 Nov 15;12:761572. doi: 10.3389/fphar.2021.761572 (PMC8634166; doi:10.3389/fphar.2021.761572)
Supplement: Supplementary file 3 [file Table1.DOCX]

Supplementary Material

# SUPPLEMENTARY TABLE S1. The primers for this whole study

| Genes | Species | Primers |
| --- | --- | --- |
| α-SMA | Rat | 5ʹ-GTCCCAGACATCAGGGAGTAA-3ʹ (forward) |
|  |  | 5ʹ-TCGGATACTTCAGCGTCAGGA-3ʹ (reverse) |
| Fibronectin | Rat | 5ʹ-ACTTCTGGTCCTCTCCCGTGTCC-3ʹ (forward) |
|  |  | 5ʹ-CGCCCTCTCCAGGAGGCTAGT-3ʹ (reverse) |
| TGF-β1 | Rat | 5ʹ-CAGGGAGTAAGGGACACGA-3ʹ (forward) |
|  |  | 5ʹ-AACAGCAGTTAGGAACCCAGAT-3ʹ (reverse) |
| IL-6 | Rat | 5ʹ-TCCGCAAGAGACTTCCAGCCAG-3ʹ (forward) |
|  |  | 5ʹ-TGTGAAGTAGGGAA GGCAGTGGC-3ʹ (reverse) |
| IL-1β | Rat | 5ʹ-GCTAGGGAGCCCCCTTGTCGAG-3ʹ (forward) |
|  |  | 5ʹ-AGGCAGG GAGGGAAACACACGTT-3ʹ (reverse) |
| Collagen I | Rat | 5′-CGCCCTCCTAATGGTCAAGG-3′ (forward) |
|  |  | 5′-AGTCTCAGGGTCCGAGGTATTC-3′ (reverse) |
| Collagen III | Rat | 5′-CGAGATTAAAGCAAGAGGAA-3′ (forward) |
|  |  | 5′-AGTACTTGGGCAGATTGACCTC-3′ (reverse) |
| MCP1 | Rat | 5′-GGCATCACAGTCCGAGTCACA-3′ (forward) |
|  |  | 5′-ACAGAAGTGCTTGAGGTGGTT-3′ (reverse) |
| TLR4 | Rat | 5′-GGCATCACAGTCCGAGTCACA-3′ (forward) |
|  |  | 5′-CTACAGACAACCACCTCAAGCACTTC-3′ (reverse) |
| TNF-α | Rat | 5′- TCAGCCGATTTGCTATCTCATA-3′ (forward) |
|  |  | 5′-AGTACTTGGGCAGATTGACCTC-3′ (reverse) |
| GAPDH | Rat | 5′- TCAGCCGATTTGCTATCTCATA-3′ (forward) |
|  |  | 5′-AGTACTTGGGCAGATTGACCTC-3′ (reverse) |
| TGF-β1 | Human | 5ʹ-GACCGCAACAACGCAATCTA-3ʹ (forward) |
|  |  | 5ʹ-AGGTGTTGAGCCCTTTC-3ʹ (reverse) |
| α-SMA | Human | 5ʹ-CACGATGTACCCTGGGATCG-3ʹ (forward) |
|  |  | 5ʹ-GCCGATCCACACCGAGTATT-3ʹ (reverse) |
| Fibronectin | Human | 5ʹ-GGAGAACAGTGGCAGAAGGA-3ʹ (forward) |
|  |  | 5ʹ-ACTCTCGGGAATCTTCTCTGT-3ʹ (reverse) |
| TNF-α | Human | 5ʹ-GAGCTGTGGGGAGAACAAAA GGA-3ʹ(forward) |
|  |  | 5ʹ-ACTCTCGGGAATCTTCTCTGT-3ʹ (reverse) |
| Collagen I | Human | 5ʹ-GAGGGCCAAGACGAAGACATC-3ʹ (forward) |
|  |  | 5ʹ-CAGATCACGTCATCGCACAAC-3ʹ (reverse) |
| Collagen III | Human | 5ʹ-ATGTTGTGCAGTTTGCCCAC-3ʹ (forward) |
|  |  | 5ʹ-TCGTCCGGGTCTACCTGATT-3ʹ (reverse) |
| IL-6 | Human | 5ʹ-TCCAGTTGCCTTCTTGGGAC-3ʹ (forward) |
|  |  | 5ʹ-GTGTAATTAAGCCTCCGACTTG-3ʹ (reverse) |
| IL-1β | Human | 5ʹ-TGATGGCTTACAGTGGCAA-3ʹ (forward) |
|  |  | 5ʹ-TAGTGGTGGTCGGAGATTCG-3ʹ (reverse) |
| TLR4 | Human | 5ʹ-ATTCCATGGCACCGTCAAGGCTGA-3ʹ (forward) |
|  |  | 5ʹ-TTCTCCATGGTGGTGAAGACGCCA-3ʹ (reverse) |
| GAPDH | Human | 5ʹ- GTCTCCTCTGACTTCAACAGCG-3ʹ (forward) |
|  |  | 5ʹ- ACCACCCTGTTGCTGTAGCCAA-3ʹ (reverse) |
