# Supplementary figures and images for: Uncovering Bupi Yishen Formula Pharmacological Mechanisms Against Chronic Kidney Disease by Network Pharmacology and Experimental Validation
Source: Front Pharmacol. 2021 Nov 15;12:761572. doi: 10.3389/fphar.2021.761572 (PMC8634166; doi:10.3389/fphar.2021.761572)

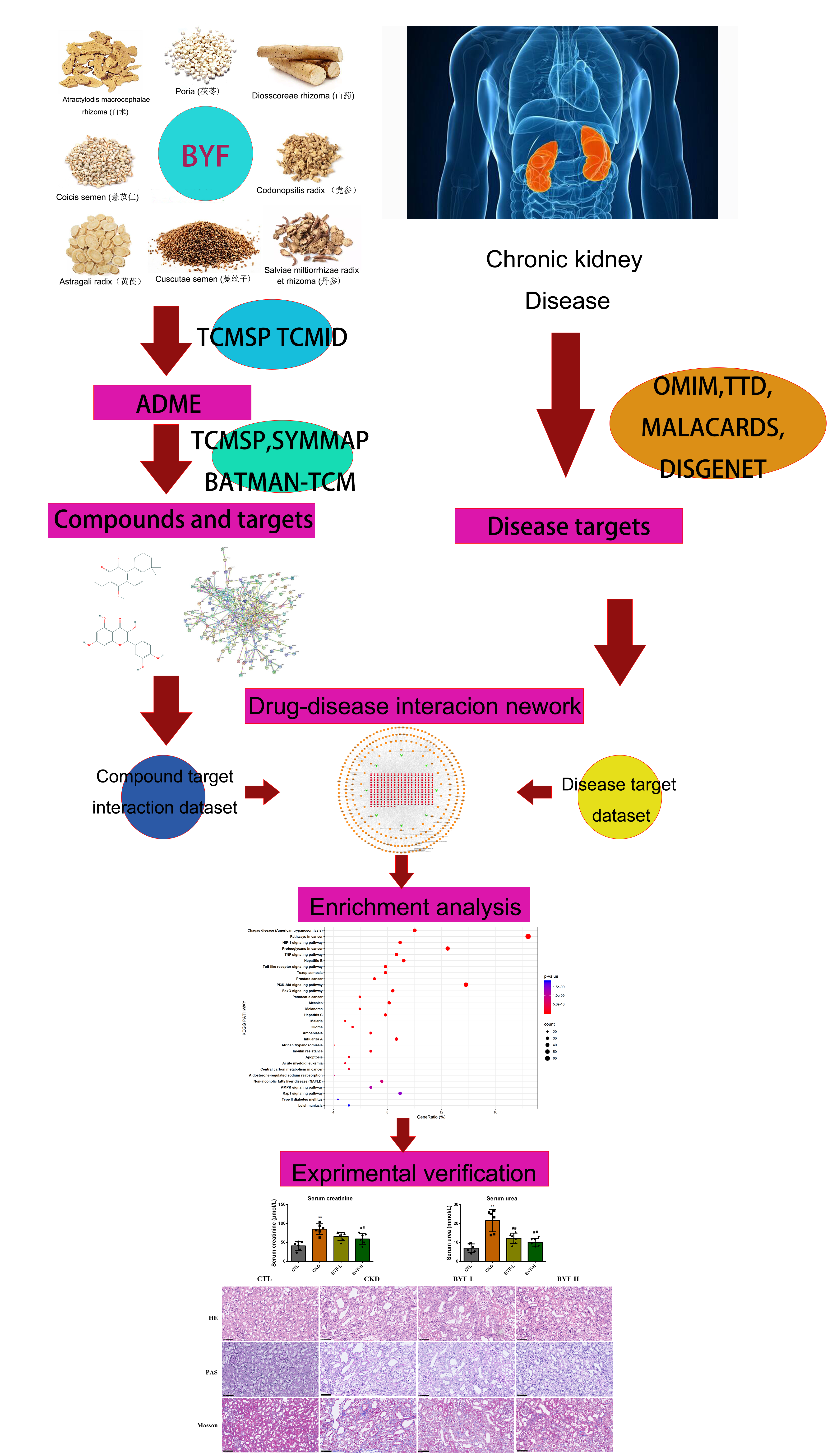

Supplement: Supplementary file 5 [file Image1.TIF]
